# Cholecystectomy vs. percutaneous cholecystostomy for the management of critically ill patients with acute cholecystitis: a protocol for a systematic review

**DOI:** 10.1186/s13643-015-0065-8

**Published:** 2015-05-30

**Authors:** Peter C. Ambe, Sarantos Kaptanis, Marios Papadakis, Sebastian A. Weber, Hubert Zirngibl

**Affiliations:** Department of Surgery II, Helios Klinikum Wuppertal, Witten-Herdecke University, Heusner Str. 40, 42283 Wuppertal, Germany; Homerton University Hospital NHS Foundation Trust, Homerton Row, London, E9 6ST UK; Department of Internal Medicine, St. Elisabeth Hospital Hohenlind, Werthmannstr. 1, 50937 Cologne, Germany

## Abstract

**Background:**

Acute cholecystitis is a common diagnosis. However, the heterogeneity of presentation makes it difficult to standardize management. Although surgery is the mainstay of treatment, critically ill patients have been managed via percutaneous cholecystostomy. However, the role of percutaneous cholecystostomy in the management of such patients has not been clearly established. This systematic review will compare the outcomes of critically ill patients with acute cholecystitis managed with percutaneous cholecystostomy to those of similar patients managed with cholecystectomy.

**Methods/design:**

Systematic searches will be conducted across relevant health databases including the Cochrane Library, Cumulative Index of Nursing and Allied Health Literature (CINAHL), MEDLINE, Embase, and Scopus using the following keywords: (acute cholecystitis OR severe cholecystitis OR cholecystitis) AND (cholecystectomy OR laparoscopic cholecystectomy OR open cholecystectomy) AND (Cholecystostomy OR percutaneous cholecystectomy OR gallbladder drain OR gallbladder tube OR transhepatic gallbladder drain OR transhepatic gallbladder tube OR cholecystostomy tube). The reference lists of eligible articles will be hand searched. Articles from 2000–2014 will be identified using the key terms “acute cholecystitis, cholecystectomy, and percutaneous cholecystostomy”. Studies including both interventions will be included. Relevant data will be extracted from eligible studies using a specially designed data extraction sheet. The Newcastle-Ottawa scale will be used to assess the quality of non-randomized studies. Central tendencies will be reported in terms of means and standard deviations where necessary, and risk ratios will be calculated where possible. All calculations will be performed with a 95 % confidence interval. Furthermore, the Fisher’s exact test will be used for the calculation of significance, which will be set at *p* < 0.05. Pooled estimates will be presented after consideration of both clinical and methodological heterogeneity of included studies. Both interventions would be compared with regard to in-hospital mortality, 30-day mortality, procedure-dependent complications, re-intervention, length of intensive care unit (ICU) stay, length of hospital stay, re-admission, and cost of treatment.

The review will be reported using the Preferred Reporting Items for Systematic Reviews and Meta-Analysis (PRISMA) statement.

**Discussion:**

This systematic review aims at identifying and evaluating the clinical value of percutaneous cholecystostomy in the management of critically ill patients with acute cholecystitis.

**Systematic review registration:**

PROSPERO CRD42015016205

## Background

Acute cholecystitis (AC) describes an acute gallbladder inflammation and is a common cause for a visit to the emergency department [1]. Although gallbladder stones are present in the vast majority of cases (calculous cholecystitis), gallbladder inflammation is possible in the absence of stones (acalculous cholecystitis). Patients usually present with pain to the right upper quadrant with fever and chills. AC is easily diagnosed using the parameters outlined in the Tokyo (TG13) guidelines. These guidelines employ information from history, physical examination, blood chemistry, and ultrasound sonography to diagnose AC. Besides providing diagnostic criteria, the TG13 guidelines enable a classification of AC in three severity grades; mild cholecystitis (grade I), moderate cholecystitis (grade II), and severe cholecystitis with organ failure (grade III) [2, 3].

Cholecystectomy, that is the surgical removal of the gallbladder, is generally accepted as the standard treatment for “fit for surgery” patients with AC [4–8]. Nowadays, the minimal invasive laparoscopic technique is the standard procedure for the management of benign gallbladder pathologies including AC. However, the management of critically ill patients with AC remains controversial. Although surgery has been employed in such patients, increased risk of perioperative morbidity and mortality due to reduced physiologic reserve is of concern. Thus, percutaneous cholecystostomy (PC), i.e., draining the inflamed gallbladder under local anesthesia, has been proposed and employed in the management of such patients, especially after failure of medical therapy [9–13].

Currently, many research groups have published their experience with PC either as a bridge to surgery or as a definitive intervention in critically ill patients with AC [11, 14, 15]. Although the results presented by some of these series appear acceptable, the small size of the study population, the retrospective study design, and possible selection bias limit their clinical value. More so, the clinical value of PC in the management of critically ill patients with cholecystitis could not be established in a systematic review by Winbladh et al. [16] in 2007. Similarly, a Cochrane review by Gurusamy et al. [17] in 2013 failed to identify the clinical significance of PC in the management of AC. This review included just 156 patients from two randomized studies and was inconclusive with regard to the clinical benefit of PC in the management of critically ill patients with AC.

In a recently published 10-year retrospective analysis, Abi-Haidar et al. [18] reported longer intensive care unit stay and hospital stay in patients managed with PC. In this series, PC was associated with higher morbidity and re-admission rates compared to CC. A similar trend was reported in a large retrospective series by Anderson and colleagues [19]. The rate of mortality for PC in their series of 43.341 patients was 61.7 %. The authors concluded, “PC may not benefit the sickest patients in whom cholecystectomy may never be considered”.

The heterogeneity of clinical presentation and comorbidities of patients with AC makes it extremely difficult to standardize management. Therefore, the clinical decision-making may not always be clear. This is especially true for elderly and critically ill patients with reduced physiologic reserve. However, modern surgical techniques as well as advances in anesthesiology and intensive care medicine could permit primary gallbladder surgery in critically ill patients.

This systematic review will compare the outcomes of critically ill patients managed with PC to those of similar patients managed with CC with the primary aim of establishing the clinical role of PC in the management of critically ill patients with AC.

### Objectives

This review will provide an overview of published data on PC and CC in the management of critically ill patients with AC. The clinical benefit of PC in the management of this subgroup of patients will be investigated by comparing the outcomes of both interventions.

## Methods/design

### Study type

We will consider all studies comparing CC and PC for the definite management of critically ill patients with AC irrespective of the presence or absence of gallbladder stones and the use of antibiotics or supportive care. Thus, studies with PC prior to CC will not be included. Since no randomized controlled trials (RCTs) exist on this topic, data for this systematic review will be acquired mainly from retrospective studies; if the question cannot be answered with certainty, this will inform the decision on whether a randomized controlled trial is needed and the number of patients required. Only studies published in English language will be included. Relevant studies in languages other than English will be considered, if an English translation is available. In such cases, a translation will be requested from the corresponding authors. Only articles published after the comprehensive availability of modern surgical techniques (minimal invasive surgery) as well as advances in anesthesiology and intensive care medicine will be included for analysis. Therefore, the search will be limited to articles published after January 1, 2000. Furthermore, all series with less than 20 participants as well as case reports will be excluded. Since a high degree of heterogeneity is expected, there will be no limits in the length of follow-up.

### Participants

All patients included in eligible studies will be considered for analysis without restriction.

### Interventions

Cholecystectomy and PC constitute the interventions to be compared.

- Cholecystectomy will be defined as the surgical removal of the gallbladder independent of the means of access, i.e., laparoscopic or open.
- Percutaneous cholecystostomy refers to the placement of a drain or a tube with the aim of draining the gallbladder content. This is usually performed under local anesthesia and image guidance via ultrasound or computed tomography.

### Primary outcomes

The primary outcomes will include in-hospital mortality, 30-day mortality, and the rate of complications. Only procedure-related complications will be analyzed since medical complications are not always procedure related.

### Secondary outcomes

- Re-intervention: any form of surgical, endoscopic or radiologic intervention following PC or CC
- Length of intensive care unit (ICU) stay
- Length of hospital stay
- Re-admission for biliary complaints
- Cost of treatment

### Search for eligible studies

Systematic searches will be conducted across relevant health databases including the Cochrane Library, Cumulative Index of Nursing and Allied Health Literature (CINAHL), MEDLINE, Embase, and Scopus using the following keywords: (acute cholecystitis OR severe cholecystitis OR cholecystitis) AND (cholecystectomy OR laparoscopic cholecystectomy OR open cholecystectomy) AND (Cholecystostomy OR percutaneous cholecystectomy OR gallbladder drain OR gallbladder tube OR transhepatic gallbladder drain OR transhepatic gallbladder tube OR cholecystostomy tube). The reference lists of eligible articles will be hand searched. Articles published after January 1 2000 will be identified using the key terms “acute cholecystitis, cholecystectomy, and percutaneous cholecystostomy.”

### Study selection

The title and abstract of each article will be screened and assessed against predetermined inclusion criteria by two independent investigators MP and SW. Each investigator must give a reason for rejecting any article. Unclear cases will be discussed with PA. A detailed full-paper assessment will be performed for each study deemed eligible for inclusion. This will be done independently and unblinded by MP, SW, and PA. Disagreements will be discussed with PA and if necessary, a fourth investigator, HZ, will be consulted. All consensuses reached must be in accordance with the protocol. The corresponding authors of eligible articles will be contacted for clarification (e.g., missing data, etc.) where necessary.

### Data extraction

A data extraction sheet designed for this systematic review by SK and PA will be used for data extraction. MP and SW will independently extract data from the included studies. PA will cross-check the extracted data. Disagreements will be resolved in all cases via discussion. If no agreement is reached, HZ will be consulted for consensus.

The following information will be extracted:

- Publication language, year, and country of origin
- Baseline features: age, sex, American Society of Anesthesiologists (ASA) score, and body mass index (BMI)
- Size of study population
- Study design
- Inclusion criteria
- Type of intervention: PC and CC
- Allocation criteria for the respective interventions
- Length of ICU stay in days
- Length of hospital stay in days
- Duration of follow-up
- Morbidity and mortality rates
- Re-intervention: type and reasons of re-intervention
- Re-admission for biliary complaints
- Cost of treatment

### Assessment of risk of bias

SK and MP will assess risk of bias of all the studies included. As per the instructions outlined in the Cochrane Handbook for systematic reviews of interventions, the Newcastle-Ottawa scale will be used to assess quality of non-randomized studies. Two previous systematic reviews [16, 17] (search to 2012) identified no randomized controlled trials directly comparing PC and CC; if an adequate number of trials are identified in our search, the analysis protocol proposed by the Cochrane Systematic Review [17] will be followed. If necessary, the corresponding authors of included articles will be contacted for further information.

### Measurement of treatment effect

Central tendencies will be reported in terms of means and standard deviations where necessary. If not reported, the standard deviation will be calculated from the standard error of the mean. The corresponding authors will be contacted whenever such data are missing. Risk ratios will be calculated where possible. All calculations will be performed with a 95 % confidence interval. Furthermore, the Fisher’s exact test will be used for the calculation of significance, which will be set at *p* < 0.05. Adjusted effect estimates will be analyzed in preference to the unadjusted estimates, using inverse-variance weighted average. Pooled estimates will only be presented after consideration of both clinical and methodological heterogeneity of included studies. If there is significant clinical or methodological heterogeneity, we will consider pooling only homogeneous studies. If this is thought to introduce too much bias, we will abandon the meta-analysis altogether.

### Unit of analysis

The unit of study will be critically ill patients with AC, both calculous and acalculous cholecystitis.

### Dealing with missing data

The responsible author will be contacted whenever possible to request for missing data. Assumption methods used to cope with missing data will be clearly outlined. If necessary, sensitivity analyses will be carried out to assess how sensitive the results will react to changes in assumption. These will be fully discussed in the review.

### Assessment of heterogeneity

The Chi square test with a *p* value of 0.05 will be used to explore heterogeneity while the quantity of heterogeneity will be assessed using *I*^2^ statistics. *I*^2^ will be interpreted according to the guidance for Cochrane reviews. It is expected that a random effects model will be used to build up the meta-analysis.

### Assessment of reporting bias

We plan to use visual inspection of the funnel plots to assess reporting bias if sufficient studies are available. We plan to use the linear regression approach described by Egger [20] to determine the funnel plot asymmetry. Possible sources of any funnel plot asymmetry will be explored since there may be publication bias but no asymmetry.

### Subgroup analysis

Subgroup analyses will be performed for the primary endpoints. These analyses will be stratified by groups of patients managed with CC vs. PC. We will also perform subgroup analyses stratified by gender, ASA score, age, and BMI if possible. In the event that stratified gender, ASA score, age, and BMI analyses are not possible, we will use meta-regression to perform analyses adjusted for the percentages of males/females, ASA scores, BMI (mean or median), and age (mean or median).

### Sensitivity analysis

Sensitivity analysis will be performed to investigate the influence of changes in the methods or data used in the individual articles on the review. This will be done for suitable features during the review process.

## Discussion

AC is a common diagnosis, and the management of critically ill patients can be challenging with high rates of morbidity and mortality. While surgery is generally offered to “fit for surgery” patients, the management of critically ill patients is unclear. Percutaneous cholecystostomy has been used in such patients after failure of medical treatment. However, this procedure has been shown to be associated with unacceptably high rates of complications and mortality.

This systematic review will give an overview of the existing data on the management of critically ill patients with AC. Directly comparing the outcomes of patients managed with cholecystectomy to those of similar patients managed with cholecystostomy may help facilitate the clinical decision-making.

## Abbreviations

AC: acute cholecystitis
ASA: American Society of Anesthesiologists
BMI: body mass index
CC: cholecystectomy
ICU: intensive care unit
PC: percutaneous cholecystostomy

## References

[1] Ambe P, Weber SA, Christ H, Wassenberg D (2014). Cholecystectomy for acute cholecystitis. How time-critical are the so called “golden 72 hours”? Or better “golden 24 hours” and “silver 25–72 hour”? A case control study. World J Emerg Surg.

[2] Sekimoto M, Takada T, Kawarada Y, Nimura Y, Yoshida M, Mayumi T (2007). Need for criteria for the diagnosis and severity assessment of acute cholangitis and cholecystitis: Tokyo Guidelines. J Hepatobiliary Pancreat Surg.

[3] Kiriyama S, Takada T, Strasberg SM, Solomkin JS, Mayumi T, Pitt HA (2013). TG13 guidelines for diagnosis and severity grading of acute cholangitis (with videos). J Hepatobiliary Pancreat Sci.

[4] Kolla SB, Aggarwal S, Kumar A, Kumar R, Chumber S, Parshad R (2004). Early versus delayed laparoscopic cholecystectomy for acute cholecystitis: a prospective randomized trial. Surg Endosc.

[5] Papi C, Catarci M, D’Ambrosio L, Gili L, Koch M, Grassi GB (2004). Timing of cholecystectomy for acute calculous cholecystitis: a meta-analysis. Am J Gastroenterol.

[6] Ambe P, Esfahani BJ, Tasci I, Christ H, Köhler L (2011). Is laparoscopic cholecystectomy more challenging in male patients?. Surg Endosc Intervent Tech.

[7] Gurusamy KS, Koti R, Fusai G, Davidson BR (2013). Early versus delayed laparoscopic cholecystectomy for uncomplicated biliary colic. Cochrane Database Syst Rev..

[8] Gutt CN, Encke J, Köninger J, Harnoss JC, Weigand K, Kipfmüller K (2013). Acute cholecystitis: early versus delayed cholecystectomy, a multicenter randomized trial (ACDC study, NCT00447304). Ann Surg.

[9] Chang YR, Ahn YJ, Jang JY, Kang MJ, Kwon W, Jung WH (2014). Percutaneous cholecystostomy for acute cholecystitis in patients with high comorbidity and re-evaluation of treatment efficacy. Surgery.

[10] Kapan M, Onder A, Tekbas G, Gul M, Aliosmanoglu I, Arikanoglu Z (2013). Percutaneous cholecystostomy in high-risk elderly patients with acute cholecystitis: a lifesaving option. Am J Hospice Palliat Med.

[11] Simorov A, Ranade A, Parcells J, Shaligram A, Shostrom V, Boilesen E (2013). Emergent cholecystostomy is superior to open cholecystectomy in extremely ill patients with acalculous cholecystitis: a large multicenter outcome study. Am J Surg.

[12] Mansour JC, Yopp AC (2014). Percutaneous cholecystostomy: the challenges of cohort analysis. J Surg Res.

[13] Ambe P, Weber S, Christ H, Wassenberg D. Primary cholecystectomy is feasible in elderly patients with acute cholecystitis. Aging Clin Exper Res. 2015;1–6. 10.1007/s40520-015-0361-0 25905472

[14] de Mestral C, Gomez D, Haas B, Zagorski B, Rotstein OD, Nathens AB (2013). Cholecystostomy: a bridge to hospital discharge but not delayed cholecystectomy. J Trauma Acute Care Surg.

[15] Sanjay P, Mittapalli D, Marioud A, White RD, Ram R, Alijani A (2013). Clinical outcomes of a percutaneous cholecystostomy for acute cholecystitis: a multicentre analysis. HPB.

[16] Winbladh A, Gullstrand P, Svanvik J, Sandstrom P (2009). Systematic review of cholecystostomy as a treatment option in acute cholecystitis. HPB.

[17] Gurusamy KS, Rossi M, Davidson BR (2013). Percutaneous cholecystostomy for high-risk surgical patients with acute calculous cholecystitis. Cochrane Database Syst Rev..

[18] Abi-Haidar Y, Sanchez V, Williams SA, Itani KM (2012). Revisiting percutaneous cholecystostomy for acute cholecystitis based on a 10-year experience. Arch Surg.

[19] Anderson JE, Inui T, Talamini MA, Chang DC (2014). Cholecystostomy offers no survival benefit in patients with acute acalculous cholecystitis and severe sepsis and shock. J Surg Res.

[20] Egger M, Davey Smith G, Schneider M, Minder C (1997). Bias in meta-analysis detected by a simple, graphical test. BMJ.

